# Isolation and test of novel yeast strains with lignin usage capability and phenolic compound resistance

**DOI:** 10.1002/mbo3.1326

**Published:** 2022-10-14

**Authors:** Anaid Bautista‐Guerrero, Rene A. Lara‐Diaz, Valérie Pihen, Erick R. Bandala, Jose Luis Sanchez‐Sala

**Affiliations:** ^1^ Department of Civil and Environmental Engineering, Engineering School Universidad de las Americas Puebla Puebla Mexico; ^2^ Department of Chemistry and Food Engineering, Engineering School Universidad de las Americas Puebla Puebla Mexico; ^3^ Division of Hydrologic Sciences Desert Research Institute Las Vegas Nevada USA; ^4^ Department of Chemistry and Biological Sciences, Sciences School Universidad de las Americas Puebla Puebla Mexico

**Keywords:** laccase, lignin degradation, pentachlorophenol, phenol, p‐nitrophenol, yeasts

## Abstract

Five yeast fungi strains (i.e., two *Cryptococcus albidus*, one *Candida guillermondii*, and two *Candida tropicalis*) were isolated from sugarcane and tested for their use of lignin as sole carbon source and their potential to grow in the presence of phenol and phenol derivatives (i.e., pentachlorophenol and p‐nitrophenol). The full set of isolated yeasts showed ligninolytic activity, achieving at least 36% lignin degradation after 25 days. The *C. albidus* JS‐B1 strain had the highest ligninolytic activity, achieving 27% lignin degradation within 4 days. This increased activity was associated with the production of ligninolytic laccase enzymes. All the tested yeast fungi strains showed growth in the presence of high concentrations of phenolic compounds (i.e., 900 mg/L phenol, 200 mg/L p‐nitrophenol, 50 mg/L pentachlorophenol) and showed significant potential for lignin and lignin by‐product degradation. Each of these five strains has the potential to be used in biological treatment processes for contaminated effluents from paper pulping and bleaching or phenol and phenol‐derivative biodegradation processes for other industrial wastewater effluents.

## Graphical abstract

*Candida* and *Cryptococcus* yeasts can degrade lignin using it as their only carbon source. *Cryptococcus* was shown to have the highest rate of lignin degradation. *Candida* and *Cryptococcus* yeasts can degrade phenols such as p‐nitrophenol and pentachlorophenol and use them as the only carbon sources. *Cryptococcus albidus* JS‐B1 shows the production of the laccase enzyme, one of the main ligninolytic enzymes.

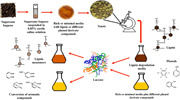

## 1 INTRODUCTION

Lignin degradation during pulp washing releases a variety of pollutants (Singh & Chandra, 2019; Zainith et al., 2019), including highly toxic, refractory phenol‐related compounds commonly associated with wastewater effluents from the petrochemical, coal mining, and coke manufacturing industries (Priyadarshini et al., 2021; Rai et al., 2021; Singh et al., 2021). Different conventional and nonconventional technologies have been tested for treating phenolic pollutants present in wastewater (Barik et al., 2021; Wei et al., 2020). Of these, biological processes are an attractive option because they require less energy and are environmentally friendly (Zdarta et al., 2021). Because of the toxicity of phenol and phenolic compounds, different microorganisms (i.e., bacteria, algae, and fungi) have been tested for their capability to degrade these compounds. Other studies have also reported the degradation of phenolic compounds in wastewater using bacteria (Bai et al., 2020; Sachan et al., 2019; Saleem et al., 2018), algae (Lindner & Pleissner, 2019; Radziff et al., 2021) and white‐rot fungi (Hou et al., 2020; L. Yang et al., 2020). Because of the amount of wastewater produced containing phenolic compounds (i.e., the pulp and paper industry consumes 200–350 m^3^ of water per ton of paper produced, and nearly 75% is discharged as wastewater), the search for new strains of microorganisms capable of degrading phenol and phenolic compounds is a significant scientific and technological task (Kumar et al., 2020).

Laccase and manganese peroxidase are well‐known lignin‐modifying enzymes generally involved in the oxidation of phenolic compounds by wood‐decomposing fungi (Fujii et al., 2020; Xia et al., 2021). The production of lignin‐modifying enzymes in certain symbiotic yeasts that can degrade phenolic compounds has also been reported (Chaijak et al., 2018). Compared to other microorganisms, only a few studies on using yeasts for phenol and phenolic compound degradation are available. Strains of the *Aureobasidium*, *Candida, Rhodotorula*, *Trichosporon*, *Geotrichum*, and *Rhodosporidium* genera have been reported to metabolize phenolic compounds as a sole carbon and energy source (Basak et al., 2014; Elena & Božena, 2001; Gupta et al., 1990; Patel et al., 2017). Yeasts also have other characteristics that provide advantages for their use in biological wastewater treatment processes, such as their rapid growth, resistance to unfavorable conditions, and ability to degrade high concentrations of phenolic compounds (up to 2000 mg/L) in a relatively short time (Fernández et al., 2017).

The goal of this study is to identify new yeast strains capable of using lignin as a sole carbon source and evaluate their growth potential in the presence of phenolic compounds. To achieve this objective, different yeast species were isolated from sugarcane, and their capabilities to produce lignin‐degrading enzymes and degrade lignin were evaluated along with their potential to use phenol and phenolic compounds as a sole carbon source.

## 2 MATERIALS AND METHODS

### 2.1 Reagents

All reagents used were reactive grade without further purification and supplied by Sigma Aldrich, USA, except where mentioned.

#### 2.1.1 Isolation of yeast from sugarcane bagasse

A 100 g batch of sugarcane (*Saccharum officinarum* L), obtained locally at the supermarket, was chopped into small pieces (about 5 × 2 cm) and air‐dried at room temperature for 3 weeks. The dried sugarcane was ground in a blender (it was previously autoclaved to eliminate potential contaminants) to obtain a fine powder (for higher substrate availability), moistened with distilled water, and kept at room temperature (25°C) in an airtight polystyrene container to reduce evaporation. Humidity was maintained for 25 days to facilitate the growth of lignin‐cellulose microorganisms. Then, 1 g of the powder was mixed with 10 mL of sterile isotonic saline solution (SISS; 0.85% NaCl) and vortexed for 5 min. From this suspension, 0.1 mL was spread on minimal medium (M9) (Kim et al., 2003) containing 2% of bacteriological agar (Bioxon®), 0.1% of alkaline lignin (Trade TCI, China) as the sole carbon source, and kanamycin (10 μg/mL) to inhibit bacterial growth. These conditions were used to favor ligninolytic microorganism growth and eliminate bacterial contamination.

#### 2.1.2 Yeast purification and identification

**Figure A1:**
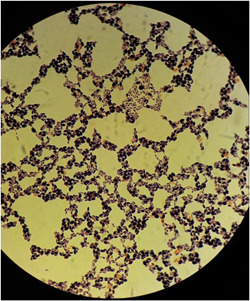
100× magnification image of yeasts under the Nikon microscope.

The different isolates from the lignin‐containing minimal medium were re‐streaked in different Petri dishes with the same medium to isolate one colony morphology. These new cultures were incubated for 5 days until medium clarification around the colonies was observed. Each colony was initially observed under a light microscope at 100× magnification (Nikon compound microscope) to confirm yeast morphology (i.e., oval/circular apiculate, elongated with a diameter of about 10 µm; see Figure A1 in Appendix A). Once the morphology of the different strains was confirmed, they were reintroduced into sterilized medium, either M9 or Sabouraud dextrose agar (SDA, Bioxon®) plus kanamycin (10 μg/mL), to obtain pure cultures. All yeast strains were grown overnight in liquid lignin degradation medium (LDM) at 35°C in a shaker bath (Lab‐Line) (150 rpm), and then mixed with 20% glycerol and stored at −80°C (Panasonic VIP PLUS ultralow‐freezer).

#### 2.1.3 Biochemical tests

For identification, each yeast isolate was subjected to the API ID 32C biochemical gallery test (Biomerieux, Ref. 32200) following the manufacturer's instructions. Briefly, five colonies were collected from a pure SDA plate culture (Bioxon®) with a bacteriological loop and suspended in SISS until tube 2 of the McFarland nephelometer reached turbidity (corresponding to about 6 × 10^8^ cells/mL according to the manufacturer's instructions). From the cell suspension, a 100 μL subsample was placed in each gallery well and incubated at 35°C for 48 h. After incubation, the gallery was read with a mini‐API reader to identify the genus and species of the different yeasts.

#### 2.1.4 Ligninolytic activity

The ligninolytic activity of the different isolated strains was measured in liquid conditions using LDM (Kim et al., 2003): M9 media plus 0.1% lignin, 5 mM MnSO_4_, 0.2% linoleic acid (LA), 2 g/L yeast extract (YE), and 0.1% carboxy‐methyl‐cellulose (CMC) at pH 7. All these components have been described to improve lignin degradation in white‐rot fungi (Cunha et al., 2010). All lignin assays were performed using the same initial culture pH and shaking (250 rpm) at 35°C. Each strain was cultured first in 10 mL of LDM for 5 days (preadaptation time). After that, cells were centrifuged (3000 rpm at 4°C) and the pellet was rinsed twice with SISS. Each strain was adjusted to McFarland nephelometer tube 2 (6 × 10^8^ cells/mL) as reported previously (Cuahtecontzi‐Delint et al., 2013). Then, after adjusting the turbidity, 1 mL was used to inoculate 125 mL of medium in 250 mL flasks with the same culture incubation conditions. Different samples (5 mL) were collected every 48 h for 18 days. Each sample was centrifuged (3000 rpm at 4°C) for 10 min. The supernatant was stored in refrigeration for lignin and ligninolytic enzyme detection, and the pellet was discharged. A flask with medium and no cells was incubated and used as a blank.

#### 2.1.5 Lignin degradation assay

Lignin concentration was measured using the diazotized sulfanilic acid reaction described in a previous report (Rajan & Srinivasan, 1992). Briefly, 10 mL of 0.5 M HCl was added to a mixture containing 0.5 g of sulfanilic acid and 0.5 g of sodium nitrite in 10 mL of 0.25 N NaOH and stirred for 30 min. Next, 10 mL of ammonium sulfamate (H_6_N_2_O_3_S) (10% w/v in water) was added to the mixture and blended for 5 min. Then, 0.6 mL of the blended mixture was placed in test tubes and 1 mL of the sample or standard solution was added to each tube. The lignin concentration was calculated according to a calibration curve with five concentrations (0.0–100 g/mL). The tubes were kept at room temperature for 30 min and then placed in a boiling water bath for 5 min. After that, the tubes were cooled to room temperature and the absorbance was read at 525 nm. All assays were performed in triplicate and the average values are reported herein. Note, ANOVA was used for the statistical analysis of all lignin degradation studies (Supporting Information: Table S1: https://doi.org/10.5281/zenodo.7023388).

#### 2.1.6 Ligninolytic enzyme activity

Ligninolytic laccase enzyme activity was measured as described in previous reports (Espina et al., 2021; Moiseenko et al., 2018; Perna et al., 2018). Briefly, the sodium tartrate buffer (100 mM) at pH 4.5 and syringaldazine solutions (0.5 mM) were prepared separately. Then, 0.4 mL of the sodium tartrate solution plus 0.1 mL of syringaldazine and 0.5 mL of the supernatant of each sample were mixed directly in a quartz spectrophotometric cell (total volume 1 mL). After gentle mixing by inversion, the absorbance at 530 nm was read on a UV‐Vis spectrophotometer (SHIMADZU UV‐1800). All the tests were carried out in triplicate and the mean values are reported herein.

#### 2.1.7 Phenol and phenolic compound sensitivity

Each yeast strain was grown separately in SDA with and without different concentrations of phenol and phenolic compounds (300 mg/L phenol [P] [Sigma Aldrich], 25 mg/L pentachlorophenol [PCP] [Sigma Aldrich], and 100 mg/L p‐nitrophenol [PNP] [Sigma Aldrich]). To assess the minimal inhibition concentration (MIC), several colonies of each strain previously grown in SDA were adjusted to ca. 6 × 10^8^ cells/mL in 10 mL of SISS (0.85% NaCl) using tube 2 (6 × 10^8^ cells/mL) of the McFarland nephelometer. The yeast suspension was used to inoculate 0.1 mL into different tubes containing 10 mL of Mueller‐Hinton broth (rich medium) with different concentrations of phenol or its derivates (300–900 mg/L of P, 10–50 mg/L of PCP [maximum water solubility 50 mg/L], and 100–500 mg/L of PNP). Tests were performed on 2 different days, each time in duplicate. Growth was recorded using an arbitrary scale of + (very low growth, few colonies visible after 96 h), ++ (low growth, colonies visible at 72 h), +++ (medium growth, colonies visible at 48 h), and ++++ (high growth, colonies visible at 24 h).

#### 2.1.8 Assessing the use of phenolic compounds as a sole carbon source

Isolated colonies from SDA containing phenol and phenolic compounds were collected from the minimal medium and re‐streaked in Petri dishes containing M9 agar medium as well as either 100 mg/L of P, 25 mg/L of PCP, or 100 mg/L of PNP. All Petri dishes were incubated at 35°C in a humidified closed box for 5 days or until growth was evident. Growth was considered positive when colony formation was observed and confirmed by microscopic observation.

## 3 RESULTS AND DISCUSSION

### 3.1 Yeast identification

The five isolated yeast strains growing on minimal medium containing 0.1% of lignin as the only carbon source were identified as *Cryptococcus albidus* (two strains JS‐B1 and JS‐B3), *Candida guillermondii* (JS‐B2), and *Candida tropicalis* (two strains JS‐B4 and JS‐B5) (The biochemical test results are shown in Table A1 in Appendix A).

### 3.2 Lignin degradation

**Figure 1:**
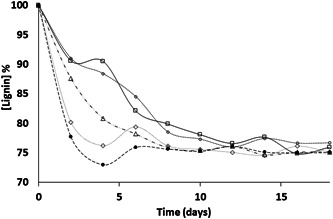
Lignin degradation by each yeast strain. *Cryptococcus albidus*, JS‐B1 (•); *Candida guillermondii* JS‐B2 (◊); *C. albidus* JS‐B3 (▲); *Candida tropicalis* JS‐B4 (□) and *C. tropicalis* JS‐B5 (○). All results show a *p* < 0.05.

Lignin degradation was assessed for 18 days in the LDM. Individual strains showed different lignin degradation activity, as shown in Figure 1. For all the experiments, the initial pH of the liquid culture medium did not show any significant change and stayed close to neutral (pH = 7.0 ± 0.2). Most of the strains showed lignin degradation activity within the same degradation percentage range (20%–25%) at the end of the incubation time (18 days). However, the highest lignin biodegradation was observed during the first 8 days, with *C. albidus* JS‐B1 showing the highest lignin degradation (27%, *p* < 0.05) during the first 4 days. The lowest lignin degradation activity (15.5%) was observed for *C. tropicalis* JS‐B5.

The lignin degradation capacity observed for all tested yeast strains agreed with other studies (Gupta et al., 1990) in which *Rhodotorula glutinis* grown in basal medium generated fast lignin degradation during the first few days followed by almost complete arrest after 4–8 days depending on the strain. One study has hypothesized that lignin concentration has an inhibitory effect on enzyme production because laccase activity decreases as lignin degradation increases (Gupte et al., 2007). The behavior observed in this study is different from typical lignin degradation using white‐rot fungi, which is usually slow but steady throughout the experiment (Knežević et al., 2013). Another possible explanation for the observed behavior is related to the bacterial batch cultures, in which rapid nutrient usage and/or metabolite production affect lignin degradation (Madigan et al., 2007). Different strains are expected to produce different enzymes or release different by‐products (Sitepu et al., 2017). In our study, *C. albidus* (JS‐B1 and JS‐B3) showed different lignin degradation rates, with JS‐B1 being faster than JS‐B3. The two *C. tropicalis* strains tested, JS‐B4 and JS‐B5, achieved similar lignin degradation. These results are consistent with another study that observed different phenotypic behavior among strains from the same species (Valderrama et al., 2003), which related the trend to enzyme expression and synthesis rate or enzyme activity associated with amino acid sequence disparities. Among all the studied strains, *C. albidus* JS‐B1 showed the highest lignin degradation (36%) followed by *C. guillermondii* JS‐B2 (~24%).

### 3.3 Laccase enzyme production

**Figure 2:**
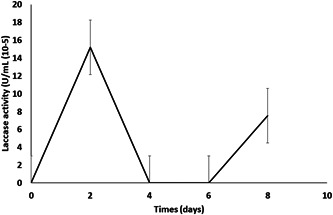
Production of laccase (Lac) by *Cryptococcus albidus*, JS‐B1.

Laccase enzyme production was evaluated for *C. albidus* JS‐B1 because that strain showed the highest lignin degradation and laccase production. Laccase activity (Figure 2) was detected starting on Day 1 for *C. albidus* JS‐B1, achieved maximum value on the second day (maintained for 4 days), and reached zero after the fifth day. Laccase activity was observed again after the sixth day and increased until reaching approximately 50% of the production achieved on the second day. Although no studies are available on ligninolytic enzyme production by yeasts, the observed trend (shown in Figure 2) is comparable to that reported for *Phanerochaete chrysosporium* (a white‐rot fungus) for which ligninolytic enzyme production, including laccase, was studied under different culture conditions (González‐Ramírez et al., 2014).

Figure 2 shows that laccase enzyme activity in the supernatant was significantly low (15.2 × 10^−5^ U/mL) compared to typical values reported for white‐rot fungi (20 U/mL) (Wang et al., 2010). This trend suggests that the laccase enzyme remains bound to the yeast membrane, as suggested in a previous study of laccase 2 (OpS5), which found that *Beauveria bassiana* generated a low supernatant concentration (Yang et al., 2017). However, no analysis was performed to evaluate a membrane‐bound laccase enzyme, so this theory deserves further exploration for demonstration.

The trend in laccase activity shown in Figure 2 agrees with the fast lignin degradation observed during the first 4 days of the experimental trials. Nevertheless, lignin degradation stopped after 8 days (Figure 1). Although more laccase was produced on Day 6, no changes in lignin degradation were observed. Other studies on ligninolytic enzymes produced by yeasts found similar results using *R. glutinis* and *Geotrichum klebahnii* (Elena & Božena, 2001; Gupta et al., 1990), in which lignin degradation occurred quickly during the first day of the experimental trial and then suddenly stopped.

### 3.4 Sensitivity to phenol and phenolic compounds

**Figure A2:**
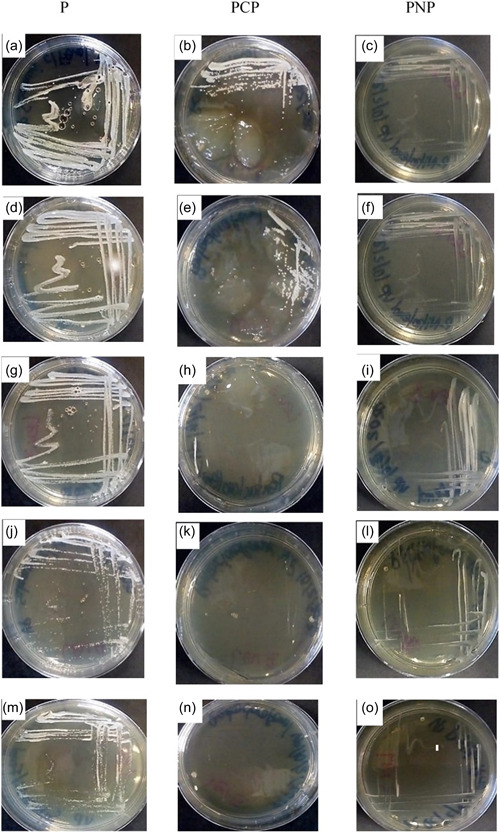
Ligninolytic yeast strains growing in Sabouraud agar media plus Phenol (P) Pentachlorophenol (PCP) and P‐Nitrophenol (PNP). *Cryptococcus albidus* (JS‐B1) (a–c); *Candida guillermondii* (JS‐B2) (d–f); *C. albidus* (JS‐B3) (g–i); *Candida tropicalis* (JS‐B4) (j–l); *Candida tropicalis* (JS‐B5) (m–o).

All the yeasts studied were tested individually and were able to grow in the presence of phenol (P) and phenolic compounds (Table 1) at concentrations as high as 900 mg/L for P, 50 mg/L for PCP, and 200 mg/L for PNP. No MIC value was determined for the test conditions used in this study within these concentration ranges of P and PCP, although strains JS‐B3, JS‐B4, and JS‐B5 showed lower growth performance after 72 h in the presence of PCP, as shown in Table 1 (Figure A2 in Appendix A). All strains were able to grow using phenol and phenol derivates as the sole carbon source. The overall growth rate followed the trend P > PNP > PCP.

**Table 1:** Growth of the different ligninolytic yeast on mycological media with phenol derivatives

Strains	S^a^ + P (300 mg/L)		S + PCP (25 mg/L)		S + PNP (100 mg/L)	
*Cryptococcus albidus* (JS‐B1)	++++	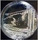	+++	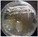	+++	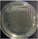
*C. guilliermondii* (JS‐B2)	++++	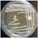	+++	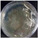	+++	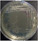
*C. albidus* (JS‐B3)	++++	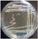	++	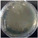	+++	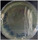
*Candida tropicalis* (JS‐B4)	++++	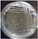	++	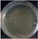	+++	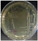
*C. tropicalis* (JS‐B5)	++++	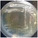	++	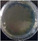	+++	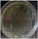

^a^ Sabouraud dextrose agar; ++++ (high growth); +++ (intermedium growth); ++ (low growth); + (very low growth).

Different yeasts with the potential to degrade phenols have been reported, and *C. tropicalis* in particular has been found capable of degrading >1500 mg/L of P (Basak et al., 2014). Fewer studies mention other species or genera (i.e., *C. guillermondii, C. albidus*) capable of degrading phenolic derivatives with higher toxicity. These findings suggest the ability of yeasts to grow in the presence of the toxic compounds normally present in industrial wastewater effluents or other sources (Aregbesola et al., 2020; Shankar et al., 2020; Yakamercan & Aygün, 2020).

## 4 CONCLUSIONS

This study successfully isolated yeast with ligninolytic activity and found they were able to grow in the presence of phenol and phenolic compounds and use these toxic compounds as a sole carbon source. The main findings are presented below:

- Yeasts of the *Cryptococcus* and *Candida* genera were isolated from sugarcane and although they were not previously reported with ligninolytic activity, here they were characterized for their capability to use lignin as the sole carbon source compared to other previously reported yeasts, such as *R. glutinis* and *G. klebahnii*.
- *C. albidus* strain JS‐B1 showed the best lignin degradation (27%) after 4 days, whereas *C. tropicalis* JS‐B5 showed the lowest activity (14%). The difference between these two species was found to be related to the laccase enzyme produced by the different strains.
- Laccase enzyme production was discontinuous throughout the first 8 days and then halted suddenly, which agrees with the results of previous studies of other strains (i.e., *R. glutinis*).
- The *C. guilliermondii* strains (i.e., JS‐B1 and JS‐B2) showed the greatest ability to grow in the presence of phenols and phenolic compounds and to use them as the sole carbon source. All the strains studied showed a significantly high MIC value (i.e., 200 mg/L).

## AUTHOR CONTRIBUTIONS

**Anaid Bautista‐Guerrero**: Conceptualization (equal); Writing, original draft; formal analysis (equal); writing, review and editing (equal). **Rene A. Lara‐Diaz**: Conceptualization (supporting); Writing, original draft (supporting); Writing, review and editing (supporting). **Valérie Pihen**: Conceptualization (supporting); formal analysis (equal); Writing, original draft (supporting); Writing, review and editing (equal). **Erick R. Bandala**: Conceptualization (supporting); Writing, original draft (supporting); Writing, review and editing (equal). **Jose Luis Sanchez‐Salas**: Conceptualization (lead); Writing, original draft; formal analysis (lead); writing, review and editing (equal).

## CONFLICT OF INTERESTS

None declared.

## ETHICS STATEMENT

None required.

## Data Availability

All data are provided in full in this paper, except for Supporting Information: Table S1: ANOVA analysis of lignin degradation by the different ligninolytic yeasts, available in the Zenodo repository at https://doi.org/10.5281/zenodo.7023388.

## APPENDIX A

**Table A1:** Standard biochemical tests result for different strains isolated from sugar cane

Test^a^	Strain JS‐B1 *Cryptococcus albidus*	Strain JS‐B2 *Candida guillermondii*	Strain JS‐B3 *C. albidus*	Strain JS‐B4 *Candida tropicalis*	Strain JS‐B5 *C. tropicalis*
0	‐	‐	‐	‐	‐
GLU	+	+	+	+	+
GLY	+	+	+	‐	‐
2KG	+	+	+	+	+
ARA	+	+	+	‐	‐
XYL	+	+	+	+	+
ADO	+	+	+	+	+
XLT	‐	+	‐	‐	‐
GAL	+	+	+	+	+
INO	‐	‐	‐	‐	‐
SOR	+	+	+	+	+
MDG	+	+	+	‐	‐
NAG	+	+	+	+	+
CEL	+	+	+	+	+
LAC	‐	‐	‐	‐	‐
MAL	+	+	+	+	+
SAC	+	+	+	+	+
TRE	+	+	+	‐	+
MLZ	+	+	+	+	+
RAF	+	+	+	‐	‐

^a^ Substrates from API gallery ID 32C:0 (none), GLU (d‐glucose), GLY (glycerol), 2KG (2‐calcium keto‐gluconate), ARA (l‐arabinose), XYL (d‐xylosa), ADO (adonitol), XLT (xylitol), GAL (d‐galactose), INO (inositol), SOR (d‐sorbitol), MDG (Methyl‐αD‐glucopyranoside), NAG (N‐acetyl‐glucosamine), CEL (d‐cellobiose), LAC (d‐lactose (bovine origin), MAL (d‐maltose), SAC (d‐saccharose), TRE (d‐trehalose), MLZ (d‐melezitose), and (d‐raffinose).
